# AuDrA: An automated drawing assessment platform for evaluating creativity

**DOI:** 10.3758/s13428-023-02258-3

**Published:** 2023-11-02

**Authors:** John D. Patterson, Baptiste Barbot, James Lloyd-Cox, Roger E. Beaty

**Affiliations:** 1 Department of Psychology, Pennsylvania State University, University Park, PA USA; 2 Psychological and Educational Sciences Research Institute, UCLouvain, Louvain-la-Neuve, Belgium; 3 Child Study Center, Yale University, New Haven, USA; 4 Department of Psychology, Goldsmiths, University of London, London, UK

**Keywords:** Automated creativity scoring, Computational creativity, Divergent thinking, Drawing assessment, Visual creativity

## Abstract

The visual modality is central to both reception and expression of human creativity. Creativity assessment paradigms, such as structured drawing tasks Barbot (2018), seek to characterize this key modality of creative ideation. However, visual creativity assessment paradigms often rely on cohorts of expert or naïve raters to gauge the level of creativity of the outputs. This comes at the cost of substantial human investment in both time and labor. To address these issues, recent work has leveraged the power of machine learning techniques to automatically extract creativity scores in the verbal domain (e.g., SemDis; Beaty & Johnson 2021). Yet, a comparably well-vetted solution for the assessment of visual creativity is missing. Here, we introduce AuDrA – an Automated Drawing Assessment platform to extract visual creativity scores from simple drawing productions. Using a collection of line drawings and human creativity ratings, we trained AuDrA and tested its generalizability to untrained drawing sets, raters, and tasks. Across four datasets, nearly 60 raters, and over 13,000 drawings, we found AuDrA scores to be highly correlated with human creativity ratings for new drawings on the same drawing task (*r* = .65 to .81; mean = .76). Importantly, correlations between AuDrA scores and human raters surpassed those between drawings’ elaboration (i.e., ink on the page) and human creativity raters, suggesting that AuDrA is sensitive to features of drawings beyond simple degree of complexity. We discuss future directions, limitations, and link the trained AuDrA model and a tutorial (https://osf.io/kqn9v/) to enable researchers to efficiently assess new drawings.

## Introduction

How can human creativity be quantified? Researchers commonly administer tests of creative thinking – spanning verbal tasks (e.g., word association) to visual tasks (e.g., sketches) – yet they are confronted with the vexing question of how to quantify creative outputs from such tests. A common approach is to ask human raters to provide subjective judgements for each response, in the spirit of the classic Consensual Assessment Technique (CAT; Amabile, 1982). Although subjective scoring can be reliable and valid (Amabile, 1982; Kaufman et al., 2007; Myszkowski & Storme, 2019), it is also time-consuming and resource-intensive, slowing the pace of research, and acting as a barrier for researchers and practitioners without the human resources to support subjective scoring methods such as the CAT. Recently, researchers have begun to rigorously test whether verbal creativity assessment can be automated using machine learning, with encouraging signs of progress, including strong correlations between computational metrics and human ratings (Acar et al., 2021; Beaty & Johnson, 2021; Buczak et al., 2023; Dumas et al., 2021; Stevenson et al., 2020). This work builds on a seminal study of automatic assessment of verbal creativity tests (Forthmann & Doebler, 2022; Paulus et al., 1970).

So far, however, computational creativity assessment has been limited almost exclusively to verbal creativity tasks. To our awareness, no extensively vetted tools for automatic scoring of *visual* creativity exist (though see Cropley & Marrone, 2022). In the present research, we aimed to address this issue by training a machine learning model on large data to automatically score responses from a creative sketching task. We examine the extent to which computational metrics correlate with human creativity ratings, and contrast those correlations to an elaboration baseline (i.e., ‘ink on the page’), which predicts human creativity ratings (e.g., Taylor et al., 2021) and would be a relatively easy heuristic for a machine to learn.

Creativity researchers often distinguish between convergent-integrative (Barbot et al., 2015) and divergent creative thinking (but see Cortes et al., 2019). Convergent-integrative thinking involves finding a single or optimal solution to a problem, such as the Remote Associates Test, which presents three words and requires finding a fourth word that relates to all three. Divergent thinking, in contrast, involves generating ideas in response to open-ended prompts, with no single or correct solution. Divergent thinking is assessed in various modalities including both verbal and figural. Verbal tasks include the alternate uses task (AUT, e.g., Guilford, 1956) – which requires thinking of creative uses for everyday objects – among other word association tasks (Benedek et al., 2012; Olson et al., 2021; Prabhakaran et al., 2014). Figural tasks, such as those from the Torrance Test of Creative Thinking-Figural (Torrance, 1972), the Test of Creative Imagery Abilities (Jankowska & Karwowski, 2015), or the Multi-Trial Creative Ideation task (MTCI; Barbot, 2018) present participants with visual stimuli (e.g., incomplete shapes) and ask them to come up with creative sketches that incorporate the stimuli.

An ongoing question in creativity research is how to score responses to such divergent thinking tasks (Reiter-Palmon et al., 2019). Historically, researchers have relied on fluency-based metrics – simply counting the number of responses produced on divergent thinking tasks – or counting the number of different categories of responses (i.e., flexibility; e.g., drawing houses or faces). However, fluency and flexibility are very highly correlated (Acar et al., 2021; Krumm et al., 2016; Said-Metwaly et al., 2020) and they do not provide information about the creative quality of responses (i.e., participants could have many common ideas), raising questions about construct validity of fluency scores as sole criterion for creativity (Silvia et al., 2008). To assess originality, uniqueness scoring has also been employed, i.e., the statistical rarity of a response in the sample. Yet uniqueness has been criticized for its sample-dependence – a response that is rare in a small sample may be less rare in a larger sample (Runco, 2008), a well-established finding related to poor reliability (Forthmann et al., 2020). It should be noted that this literature is based largely on verbal and not figural creativity. Nevertheless, the influence of dataset size on rarity should be expected to hold across modalities.

Another approach to scoring responses to divergent thinking tasks is subjective creativity scoring (Silvia et al., 2008). Subjective scoring was inspired by the CAT of real-world creative products (e.g., poems, inventions), where “experts” provide their subjective evaluations of creativity based on a loose set of criteria. Consistent with the tenets of Classical Test or ‘true score’ theory – which assumes that observed data consist of a true underlying value plus random error – evaluations are most commonly averaged across experts to estimate the creativity of a given product (or modeled as a latent variable capturing the shared variance across raters). However, more contemporary latent variable approaches like Judge Response Theory (JRT) – an application of Item Response Theory to rating data – model evaluations as an underlying latent construct not only driven by the creativity of the product, but also the properties of the judges/raters (Myszkowski & Storme, 2019). When applied to divergent thinking tests, subjective scoring typically involves training raters to rate the originality of responses, using a continuous or ordinal scale (e.g., Silvia et al., 2008, 2009). Subjective scoring of divergent thinking responses has shown evidence of reliability and validity, including positive correlations with measures of real-world creative behavior and achievement (Jauk et al., 2014; Said-Metwaly et al., 2022).

Although subjective scoring and the CAT have their merits, they also have practical limitations for conducting creativity research, including labor cost and subjectivity. Regarding labor cost, researchers must train a team of careful and consistent raters, who then have to rate thousands of responses to divergent thinking tasks. Many researchers – as well as practitioners administering creativity tests to students – do not have access to the human resources required to score large volumes of creative responses, constituting a significant barrier for administering creativity assessments. Moreover, the rating process is time-consuming, leading to issues of rater fatigue, which adversely impacts reliability and validity of scores (Forthmann et al., 2017). Regarding subjectivity, rater agreement can fluctuate dramatically from study to study, and can vary based on rater characteristics (e.g., personality; Ceh et al., 2022; Tan et al., 2015) – another source of noise that adversely impacts the psychometric properties of creativity assessments.

To address these challenges, researchers are exploring whether tools from machine learning can automate creativity assessment (Acar et al., 2021; Beaty & Johnson, 2021; Dumas et al., 2021). In the verbal domain, distributional semantic models have been applied to calculate semantic distance – the cosine distance between word vectors, based on their co-occurrence in large text corpora. Semantic distance captures notions of novelty and remoteness: words that tend to co-occur frequently in language have a low semantic distance compared to words that tend to co-occur infrequently. Several studies have examined the reliability and validity of semantic distance for verbal creativity measurement. For example, Beaty and Johnson (2021) and Dumas et al. (2021) found that semantic distance correlates strongly and positively with human creativity ratings. Semantic distance scores also predict external indicators of creativity, such as personality (i.e., openness to experience) and real-world creative achievement, highlighting its predictive validity and promise for addressing the limitations of subjective creativity scoring.

However, automated scoring methods in the visual domain of creativity – arguably the most canonical form of creative expression (Morriss-Kay, 2010) – have lagged behind their verbal counterparts. Visual creativity tests commonly present participants with open-ended prompts, such as incomplete shapes (e.g., Torrance, 1972), and ask them to provide an original sketch. Importantly, most visual creativity tests do not require artistic competence or technical proficiency; they tend to emphasize the originality of the ideas expressed, usually in the form of simple line drawings. Recently, Barbot (2018) introduced a tablet-based drawing task, which presents participants with incomplete shapes and asks them to make creative sketches that incorporate the shapes. Barbot’s tablet task offers a significant extension of paper-and-pencil tests by capturing rich reaction time (RT) data, which provide automated metrics of fluency that correspond to distinct phases of the drawing process (e.g., exploration: the elapsed time from prompt presentation to initial contact with the screen; Barbot, 2018). However, the task does not have an automated way to calculate the originality of the drawings – it can only provide RT data on fluency, not drawing quality – requiring manual/subjective creativity scoring by human coders.

At present, we are unaware of any extensively validated approaches for automated visual creativity assessment. In fact, we are aware of only one (recently introduced) approach for scoring the Torrance Test of Creative Thinking – Drawing Production task (TCT-DP; Urban, 2004) via an artificial neural network (Cropley & Marrone, 2022). In their study, the authors trained and tested a convolutional neural network (CNN) to classify TCT-DP drawing quality based on a total of 414 drawings. Although a promising approach, there are limits to the method employed by Cropley and Marrone (2022) that provide motivation for the present work. For one, the model only targeted the TCT-DP. Yet, there are a diversity of visual creativity tasks (including the MTCI; Barbot, 2018) that Cropley and Marrone’s (2022) approach do not address. Second, Cropley and Marrone’s model categorizes responses into different quality bins with no intermediate values between bins – unlike neural networks with a regression (instead of a classification) output layer which can output intermediate values even if trained only on ordinal targets. The issue with bins and the classification output layer, which the authors identify themselves, is that responses with very different degrees of creative quality can be treated as the ‘same’ (Cropley & Marrone, 2022). Third, from a machine learning perspective, the dataset (414 drawings; ~ 60 test drawings) used to train and evaluate Cropley and Marrone’s (2022) model is fairly small. While Cropley and Marrone used best available practices for training neural networks under conditions of small data (e.g., transfer learning; C. Tan et al., 2018), additional data is often desirable for training a model and evaluating its generalizability. Finally, the model trained by Cropley and Marrone (2022) was not released publicly, and no other method for automatically scoring visual creativity is currently accessible to the research community. In the present work, we develop an open-access, continuous-output, automated creativity assessment platform using big data to train and vet our approach.

## The present research

Computational creativity assessment offers powerful methods to automate and accelerate creativity research, with applications from education to industry. However, well-validated, automated tools are currently only available in the verbal domain (e.g., semantic distance) – with limited options in the visual domain. Here, we developed and tested a machine learning model – Automated Drawing Assessment (AuDrA) – to automatically predict the creative quality of individual sketches on a visual divergent thinking task. Leveraging contemporary approaches to computer vision, we trained a modified ResNet – a deep convolutional neural network (He et al., 2016) – to estimate human creativity judgments and evaluated its capacity to generalize to new sketches using a combined total of over 13,000 sketches drawn from the MTCI digital drawing task of Barbot (2018). In the task, participants start with an abstract or concrete shape on the screen and are asked to draw atop it in the most creative way they can think of, in a self-paced manner, while incorporating the starting shape into their drawing.

We first conduct a computational experiment to find the optimal model that minimizes mean squared error (MSE) and, later, maximizes Pearson correlation on the validation dataset (detailed further below). After training the optimal version of AuDrA on 70% of the primary dataset of 11,000 sketches – rated by 50 human raters using a planned missing design – we then test the model on the held-out data to assess its predictive accuracy and generalizability, assessing it on both MSE and global correlation between model predicted and actual ratings (i.e., on response-level data). Finally, we test the limits of AuDrA’s generalizability via additional datasets that span the near-to-far generalization continuum. These datasets range from new drawings rated by new raters on the *same* drawing task (i.e., creating sketches using incomplete shapes; Barbot, 2018) to drawings resulting from a different drawing task altogether (i.e., creating sketches using recognizable, concrete objects as a starting point). At each step, we assess AuDrA’s correlation with human ratings at the response level and, as vital evidence of AuDrA’s incremental validity, compare with the correlation between degree of elaboration (i.e., ‘ink on the page’) and human ratings, given that elaboration is a simple metric that correlates with human ratings on numerous creativity tasks (e.g., Taylor et al., 2021) and could be fairly easily “learned” by machine learning algorithms (i.e., “more ink is more creative”).

## Method

The rating datasets, trained AuDrA model (along with a tutorial), and analysis scripts are all accessible on OSF (https://osf.io/kqn9v/).

### Datasets

This work employed four distinct datasets of rated drawings – totaling over 13,000 drawing–rating pairs. Though all four datasets resulted from experiments that employed the same general procedure (Barbot, 2018), the datasets differed with respect to the starting stimuli: the starting stimuli were either *abstract* (which did not form a closed object; the ‘incomplete shapes’ task) or *concrete* (which formed recognizable objects, like sunglasses; the ‘object transformation’ task). Three of the four datasets (~ 95% of the 13,000 total drawings) were based on the abstract version of Barbot’s (2018) drawing task. Thus, we focused on training the model to perform on the abstract drawing task in order to optimize its predictive capacity for that specific task – though we also did test whether creativity score predictions from our model trained on abstract drawings could generalize to concrete object drawings. The breakdown and attributes of the four datasets are detailed immediately below.

The primary dataset (*n* = 11,075 drawings) was used with the purpose of establishing the canonical train/validation/test split, following best standard practices in machine learning (Zhou, 2021). We employed a 70/10/20% split for training, validation, and test, respectively. Drawings and corresponding ratings from the primary dataset were randomly assigned to one of the subsets (i.e., train, validation, or test). To be clear, the training subset is the data the model gets to learn from, the validation subset is used to determine the best settings for the model, and the held-out test subset is used to assess model generalizability only once the best fitting model settings are obtained. The primary dataset was developed and rated specifically with the purpose of deriving a machine learning model capable of automated predictions. Rather than using the standard three or four raters, as is common in creativity assessment, we obtained ratings from 50 raters in order to provide stable (i.e., approaching the population mean) ratings for the model to train on. Interrater reliability for each of the splits was assessed using a two-way random effects intraclass correlation coefficient model of average consistency (ICC[C,k]), via the ‘irrNA’ package for R (Brueckl & Heuer, 2022). Reliability for the training (ICC = .89; 95% CI [.89, .9]), validation (ICC = .9; 95% CI [.89, .91]), and test (ICC = .9; 95% CI [.89, .9]) subsets were all high. The 50 raters (*M*_Age_ = 19.4; 60% identifying as female) rated the primary dataset using a planned missing data design (Graham et al., 2006). The raters were trained undergraduate students recruited from Penn State University who received partial course credit for their participation and completed informed consent. Raters were instructed to rate the creativity of the idea expressed in the drawing, not the technical proficiency of the drawing. They rated each drawing using a 1 (*not at all creative*) to 5 (*very creative*) scale (Forthmann et al., 2019). When making their ratings, the raters also viewed the respondents’ (i.e., the people who made the drawings) descriptions of what they drew, but the labels/descriptions were *not* used by the model for its training or when making creativity predictions in this work. Full rater instructions and training materials are available on OSF (https://osf.io/kqn9v/); below is a sample of the instructions:

“*During this task, you will rate how creative you think a set of simple drawings are. The drawings were created by a range of people (adults, children, from different countries). Their task was to create the most original drawing they could think of from a simple abstract shape that had to be used as a part of the drawing.*

*You will be shown each drawing together with a label that will help you identify what was drawn. For each drawing, rate the creativity on a 1 to 5 scale, with 1* *being "not at all creative", and 5 being "very creative". Try not to rate how artistic or pretty the drawings are, but how creative the ideas are*.”

The three additional datasets were used to test the limits of AuDrA’s ability to generalize to new raters and tasks. *Rater generalization dataset 1* consisted of 670 new drawings from the abstract, incomplete shapes task (the task the model was trained on) and was rated by three new raters (ICC = .73; 95% CI [.69, .76]). *Rater generalization dataset 2* was composed of 722 drawings that resulted from the abstract, incomplete shapes task and was rated by six raters (ICC = .9; 95% CI [.89, .91]). Finally, the *rater and task generalization dataset* consisted of 679 drawings (resulting from the concrete ‘object transformation’ task, which uses a recognizable concrete object as a starting image, as opposed to the abstract figure in the incomplete shapes task) and was rated by three raters that evaluated that dataset (ICC = .63; 95% CI [.58, .68]).

The differences between these datasets offer alluring opportunities to test the generalizability of our modelling approach. Specifically, these datasets allow us to test the extent to which the model can generalize to: (1) drawings that the model was not trained on, (2) drawings the model was not trained on that were also rated by raters it was not trained on; and (3) drawings the model was not trained on that resulted from a distinct drawing task (i.e., concrete object starting images of the transformation task) *and* that were rated by raters the model was not trained on. Thus, the datasets are situated on a continuum based on the degree of generalization required to succeed at creativity score prediction.

### Model choice and modification

There exists a multitude of computer vision approaches based on the CNN and visual transformer frameworks (Bi et al., 2021; Dosovitskiy et al., 2021; He et al., 2016) that may be suitable for creativity score prediction. In the present work, we opted to use ResNet (He et al., 2016), a CNN, as the computer vision backbone for AuDrA. Though no longer the state-of-the-art, ResNet is often appealed to as a baseline in machine learning research due to its excellent accuracy to model size ratio (Canziani et al., 2017), and its enhanced generalization ability over standard feedforward neural networks (Huang et al., 2020). In other words, the model architecture is able to do “more with less,” relative to many other convolutional neural networks. As neural networks can be rather large – containing millions or billions of parameters – selecting a model that is performant but also compatible with computational resources researchers are likely to have is key for making AuDrA as accessible as possible.

**Fig. 1:**
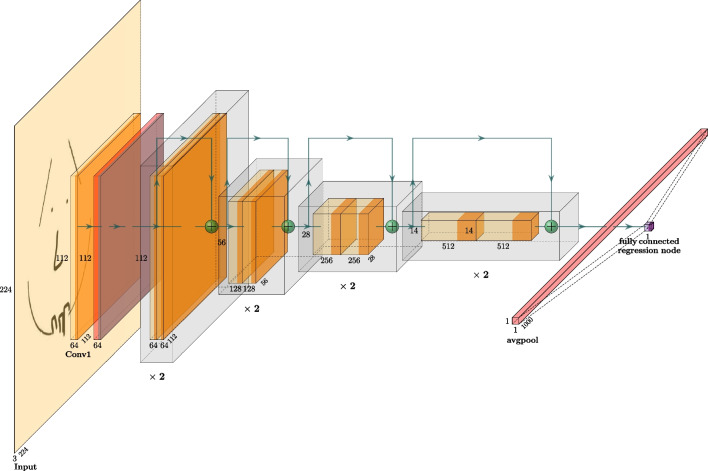
AuDrA model architecture, based on the ResNet-18 Architecture. The model consists of initial convolution and max pooling layers followed by four stages (shown as transparent gray rectangular prisms). Each stage includes four convolutional layers. Skip connections – shown as green ‘hopping’ arrows that go from the beginning of each stage to the end – add the stage’s input to its output (shown as spherical green addition signs at the end of each stage). This focuses the model on learning the difference (i.e., residual) between the output and input of the function (i.e., stage). After the final stage, model activations are pooled and flattened to a 1k dimension activation vector that is densely connected to a single regression output node (purple cube) that produces AuDrA’s creativity prediction. For greater detail on the ResNet architecture, see He et al. (2016)

Though we opted to use ResNet in the current work, it is important to note that ResNet is not directly extensible to the issue of creativity score prediction. This follows from the fact that ResNet is geared toward image classification. In other words, ResNet, once trained, answers the question of ‘what is it?’ (e.g., is it a Bird? A Chair? A Building?), like the visual creativity model of Cropley and Marrone (2022). Accordingly, ResNet has an output layer which yields a predicted probability distribution across the category labels it was trained on (e.g., Bird: 95%, Chair: 3%, Building: 2%). To tailor the model to the problem of creativity prediction on a continuous scale, we swapped ResNet’s *n*-category output layer with a single regression output node. By doing this, it transformed ResNet from a categorical, ‘what bin is it?’ model to a continuous creativity prediction model. The scalar value produced by AuDrA’s single regression output node corresponds to its creativity assessment (on a scale of 0–1.0) for the input item. Figure 1 presents the AuDrA architecture.

### Computational experiment

CNNs have different hyperparameters (i.e., free parameters in cognitive science lingo), including extra-model parameters (e.g., how many passes through the data should occur during training), that can be adjusted to optimize predictive accuracy. We searched over five potentially relevant hyperparameters in a computational experiment – using performance (mean squared error and, later, Pearson correlation) on the validation dataset to assess which hyperparameter settings were best. First, we compared two different variants of ResNet – the 18 and 34 layer versions. While deeper networks have theoretically higher capacity to represent more information by virtue of their larger number of weights and ability to compose more complex visual features, recent work suggests that deeper is not always better and smaller networks may yield better performance on some learning problems (Tan & Le, 2019). Second, we searched over learning rate – i.e., the degree to which the weights of the model are updated on each batch of training images. Larger learning rates correspond to larger changes to the model weights each batch. Using too small of a learning rate can mean the model trains slowly and never reaches a good solution within the training time allotted. Too high of a learning rate can cause instability during training, such that the model never converges on a solution or gets progressively worse at the target problem. Based on piloting, we found good validation set performance with a value of 1e^-5^, so we expanded the range to include possible learning rate values in the range of 1e^-7^ to 1e^-4^. Third, we varied the state of the model’s weights before the model was trained on the drawings dataset. Specifically, we varied whether the weights of the model’s body (i.e., all weights except the newly added regression head) were randomly initialized or set to values obtained from training the model on the ImageNet 1k dataset (Deng et al., 2009). To be clear, we did not pre-train the models on the ImageNet 1k dataset ourselves. Instead, we used the pre-trained ImageNet 1k weights available via the PyTorch package (Paszke et al., 2019) for each model variant (ResNet-18 or -34). As the ImageNet dataset consists of over 1,281,167 training images, each which belongs to one of 1000 non-overlapping real-world object concepts, we pursued this hyperparameter based on the assumption that structure present in images of real-world objects may transfer well to drawings (Hendrycks et al., 2019) – to the extent that sketches recapitulate the structure present in real-world objects. It is important to note that using the ImageNet pre-trained weights as the starting point for training on the drawings dataset is an instance of ‘transfer learning,’ a machine learning approach that can be used to maximize model performance by adapting knowledge learned from one domain or problem to another domain or problem (for a review see Tan et al., 2018). Transfer learning can be particularly helpful in cases where insufficient training data are available. Fourth, we searched over batch size (i.e., the number of images the model gets accuracy feedback on at a time). Larger batch sizes lead to faster model training, but this can come with the tradeoff of slightly poorer generalization capacity for the fully trained model (Masters & Luschi, 2018). For this reason, we looked at two mini-batch sizes that tend to perform well (16 vs. 32 items). Finally, we searched over the number of training epochs (i.e., the number of full passes through the training dataset).

To manage the hyperparameter search process during the experiment, we used a Tree Parzen Estimator (TPE). TPEs use Bayesian logic to select the most promising hyperparameter values by initially assuming a uniform prior over hyperparameter settings but, as the TPE gains more information about how different hyperparameter setting combinations relate to the model’s performance, it gets better at selecting settings that are most likely to maximize accuracy. The performance metric we aimed to minimize in our computational experiment via the TPE was mean squared error between the model-predicted and human-provided ratings, at the individual response/drawing level. We ran the TPE for 505 hyperparameter selection iterations – that is, 505 Bayesian-selected values of model depth, learning rate, ImageNet pretraining, and training batch size. After the TPE search was completed, we searched for the number of training epochs that maximized the Pearson correlation between individual human and model-provided ratings (again, using the validation dataset).

### Dataset preprocessing

**Fig. 2:**
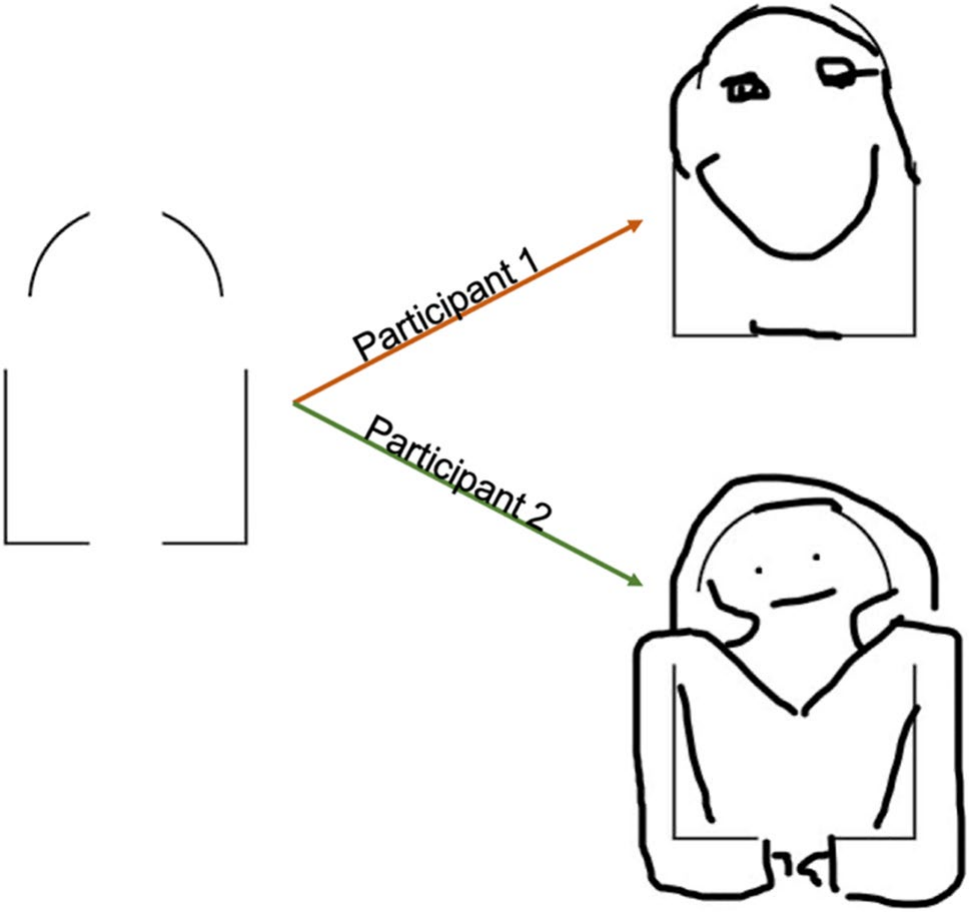
Example of an MTCI Incomplete Shapes Task item and responses*.* On the left is one of the starting images from the ‘incomplete shapes’ task. On the right are products from two participants

To prepare the data for training and test, we preprocessed both the drawing images (the model’s inputs) and human-provided creativity ratings (the model’s targets) in all datasets. To prepare the drawings – which were black ink on white background as seen in Fig. 2 – we first inverted the color scheme. In grayscale RGB color space, white is represented by high values and black is represented by low values. As we wanted the metrics to focus on predicting positive space (ink) and not negative space (background), we inverted the RGB values such that ink was represented by high values. This facilitated interpretability of our elaboration metric, such that ink on the page would correlate positively with human ratings (as is commonly observed in the creativity literature; e.g., Taylor et al., 2021). We also followed the standard practice of standardizing each image from the generalization datasets (i.e., validation, test, rater generalization 1 and 2, and far generalization) – on a channel-wise basis (i.e., RBG) – by the mean and standard deviation of the training set’s channel activations. To be clear, this standardizes datasets according to degree of elaboration within whole images irrespective of spatial location (i.e., not on the individual pixel level). Also note, in grayscale images the mean is identical across channels and so is the standard deviation. Lastly, we performed image resizing on all input images such that they fit the required input dimensionality of ResNet (224 × 224 pixels by three channels, RGB).

To prepare the human creativity ratings (i.e., the model’s target values) for the computational experiment, in each dataset individually (i.e., primary, rater generalization 1, rater generalization 2, and far generalization), we first z-scored the totality of each rater’s ratings across items (to adjust for rater severity; Long & Pang, 2015). Z-scored values for each item were then averaged across raters. This yielded a composite rating for each sketch – a common practice used in procedures inspired by the CAT (Barbot et al., 2019), and one that may be particularly important given our use of a planned missing design for the primary dataset, in which raters rate few items. The composite ratings were then min-max normalized to a 0–1 scale, where 1 is maximally creative. This was done to shift the negative values from the z-score scale to be zero or greater, as the model’s output node employs a rectified linear unit activation function – the outputs of which are always zero or greater.

## Results

### Computational experiment

The top five hyperparameter combinations found can be seen in Appendix Table 2. The results of the computational experiment revealed that the best fitting model – the one that minimized mean squared error between model-predicted and human ratings – was one that (1) used the shallower 18-layer ResNet architecture, (2) was pre-trained on the ImageNet dataset prior to learning to predict sketch creativity, (3) employed a mini-batch size of 16 images, and (4) used a learning rate 3.466e^-4^. With the best fitting model, we then searched for which epoch (between 1 and 150) maximized the Pearson correlation between human and model-predicted ratings on the validation set; we found 136 epochs maximized correlation magnitude.

After obtaining the best fitting model, we re-trained and tested the model on judge response theory (JRT; Myszkowski & Storme, 2019) theta scores, instead of the mean z-scored human ratings used in the computational experiment. The rationale is that, because JRT theta scores estimate a latent factor that accounts for the underlying creativity level for each drawing (i.e., controlling for raters’ inaccuracy and measurement error), they may augment AuDrA’s predictive accuracy. To this end, we used a Graded Response Model from the JRT package for R (Myszkowski, 2021) to compute theta scores for the creativity ratings in all of our datasets before then range normalizing the theta scores of each dataset individually to a 0–1 scale (the scale the model expects). To be explicit, we computed theta scores on an individual dataset basis (i.e., ratings for the primary, rater generalization 1, rater generalization 2, and far generalization sets were transformed to theta scores independently). We decided to compute theta scores on a dataset-to-dataset basis – instead of merged into one large dataset – for two reasons. First, the raters were not shared across datasets in most cases. Thus, merging all datasets into one and computing theta scores for the combined dataset would treat different raters (who likely have different biases) as the same rater, invalidating the approach. Second, computing theta scores based on a merged dataset would allow properties of the primary dataset (including the training set) to influence the theta scores generated for the other datasets – a form of data leakage that holds the potential to affect performance on the generalization datasets.

Informally, we found the theta scores to have a net-neutral effect – adding ~ 1–2% performance for some measures and reducing performance by roughly the same amount for others. However, given the theoretical appeal of using scores that tap the underlying construct of creativity, we retain the JRT-based approach in the analyses that follow. Plots of conditional reliability – i.e., rater reliability across different degrees of creativity – from the JRT package for each assessment dataset are found in Appendix Fig. 9.

### Primary dataset analyses

The primary dataset constituted 11,075 sketches that were divided into training (70%), validation (10%), and held-out test (20%) subsets. Because performance on the training subset says little about the model’s abilities – CNNs can attain perfect accuracy on the items they were trained on, but fail to generalize to untrained inputs – we focus the analysis on sketches the model was not trained on (i.e., the validation and test subsets). However, the rating distribution for the training dataset can be found in Appendix Fig. 10.

#### Validation

The validation set consisted of 1104 sketches. Model performance was evaluated on the validation subset after each training epoch. Using these data, changes to AuDrA’s predictive capacity can be tracked across the time course of the 136-epoch learning period. If the model is acquiring properties of sketches that are effective for predicting creativity, performance should improve across epochs. To track performance, we computed the Pearson correlation between model-predicted and human-provided ratings for each of the 136 training epochs (i.e., the optimal number of epochs according to the computational experiment).

As an added baseline measure, we computed the correlation between the number of inked pixels in the sketches and human creativity ratings. This serves two purposes. For one, indicators of elaboration have been shown to be positively related to creativity ratings (e.g., *r* = .41; Beaty & Johnson, 2021; Forthmann & Doebler, 2022; Paulus, 1970; Taylor et al., 2021). Second, it provides a degree of insight into *what* AuDrA is learning. If AuDrA’s performance is comparable to the baseline measure, it would suggest AuDrA merely learns a “more ink is more creative” rule. Alternatively, if AuDrA’s performance exceeds the baseline measure, it would suggest that it is acquiring knowledge of figural properties of sketches that are associated with creativity ratings.

**Fig. 3:**
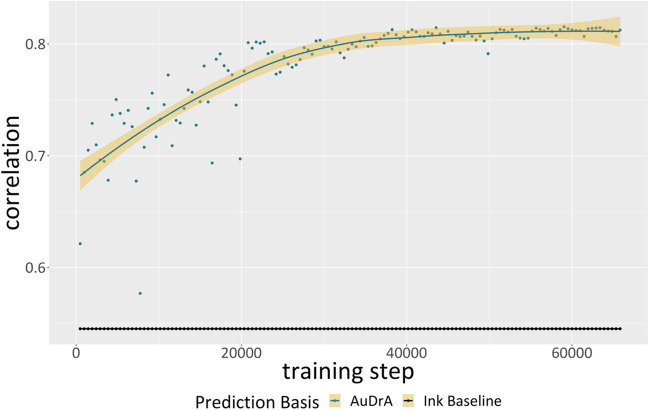
Model–human correlation on the validation set across training epochs. Each dot corresponds to the correlation between model-predicted creativity ratings (blue) or amount of ink on the page (black) and human creativity ratings. The blue line shows the line of best fit (Loess curve) across training steps for the correlation AuDrA achieved with human ratings. The yellow bands show the 95% confidence intervals for the line of best fit across the time course of training, not the momentary 95% confidence interval for the AuDrA-human correlation at each time point, given that each time point has only one observation

AuDrA’s validation performance across epochs is depicted in Fig. 3. As can be seen, correlations with human ratings became stronger as training progressed. AuDrA successfully learned to predict human ratings, achieving a high degree of accuracy (MSE = .009), and reaching asymptotic performance above *r* = .80. Importantly, while degree of elaboration correlated strongly with human creativity ratings (*r*_*Ink*_ = .53; 95% CI [.49, .57]), AuDrA’s correlation with human ratings was much larger (*r*_*AuDrA*_ = .81; 95% CI [.79, .83]). This suggests AuDrA did not simply learn that “more is more creative” but, instead, that AuDrA developed more sophisticated knowledge about how visual features relate to creativity. Notably, the prediction accuracy was computed at the level of individual drawings, not the aggregated level of participants (i.e., averaged across multiple drawings), which should be expected to be even higher.

While AuDrA’s predictions strongly correlated with human ratings, the coefficient says little about *where* the model tends to perform well and where it does not – is AuDrA equally accurate, no matter the creativity level of the product? Or does AuDrA’s accuracy differ as a function of how creative humans evaluate a given drawing? For example, AuDrA may be better at predicting less creative drawings than more creative drawings (according to human ratings).

**Fig. 4:**
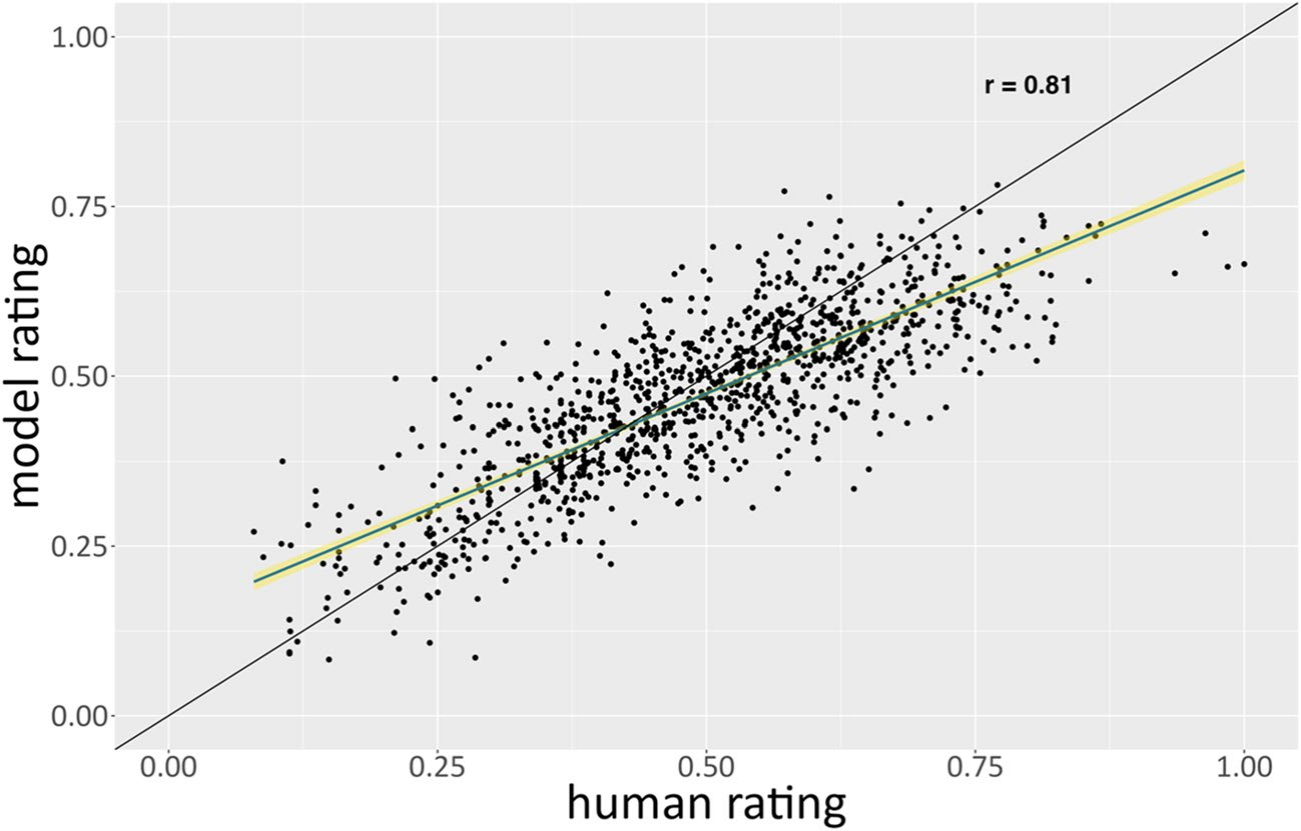
Validation set: Predictive accuracy by rating magnitude. Each dot corresponds to a particular sketch from the validation subset of the primary dataset. The *y*-axis shows the value predicted by AuDrA whereas the *x*-axis shows the creativity value provided by human raters. The diagonal black line reflects ideal performance (i.e., *r* = 1), where greater deviations from the line reflect poorer predictive accuracy. The blue line reflects the line of best fit with 95% confidence intervals in yellow

To examine this qualitatively, we plotted AuDrA’s predictions against human creativity ratings (Fig. 4). Interestingly, AuDrA performed best on products that were evaluated by humans as being moderately creative (i.e., between .3 and .6 on the 0–1 scale) but displayed larger prediction errors for items that were either the least or most creative. This may be due to lower conditional reliability in the validation set at the low and high end of the rating spectrum (Appendix Fig. 9) or simply due to the fact that the model received very little supervision at the tails of the rating distribution during training (Appendix Fig. 10). Irrespective of the cause, the nature of these prediction errors differed as a function of whether the drawing was on the high or low end of the creativity spectrum. For products deemed the least creative by humans, AuDrA tended to *overestimate* how creative they were. Conversely, on the opposite side of the spectrum, AuDrA also tended to *underestimate* the creativity of products humans deemed most creative.

#### Test

An important consideration is that the computational experiment to find AuDrA’s best settings was based entirely on maximizing performance on the validation subset’s drawings. In this way, there is a risk that performance on the validation subset may reflect ‘teaching to the test’ more than AuDrA’s ability to generalize to new images. Critically, the held-out test set, consisting of 2216 drawings, had no influence on AuDrA’s final form and serves as a more stringent test of AuDrA’s abilities to generalize to a new set of drawings.

Using this test subset, AuDrA’s predictions closely fit the human ratings (MSE = .009). We again computed the correlation between AuDrA’s predictions and human-judged creativity, and compared that to the correlation between degree of elaboration and human ratings. With this larger sample of held-out sketches, AuDrA’s predictions achieved a very strong correlation with human creativity ratings (*r*_*AuDrA*_ = .80; 95% CI [.79, .82]), with minimal loss of performance relative to the validation subset. Importantly, AuDrA’s predictive accuracy dramatically outpaced that of elaboration (*r*_*Ink*_ = .41; 95% CI [.37, .44]), suggesting AuDrA developed a nuanced understanding of predictive visual features above and beyond the amount of ink on the page.

**Fig. 5:**
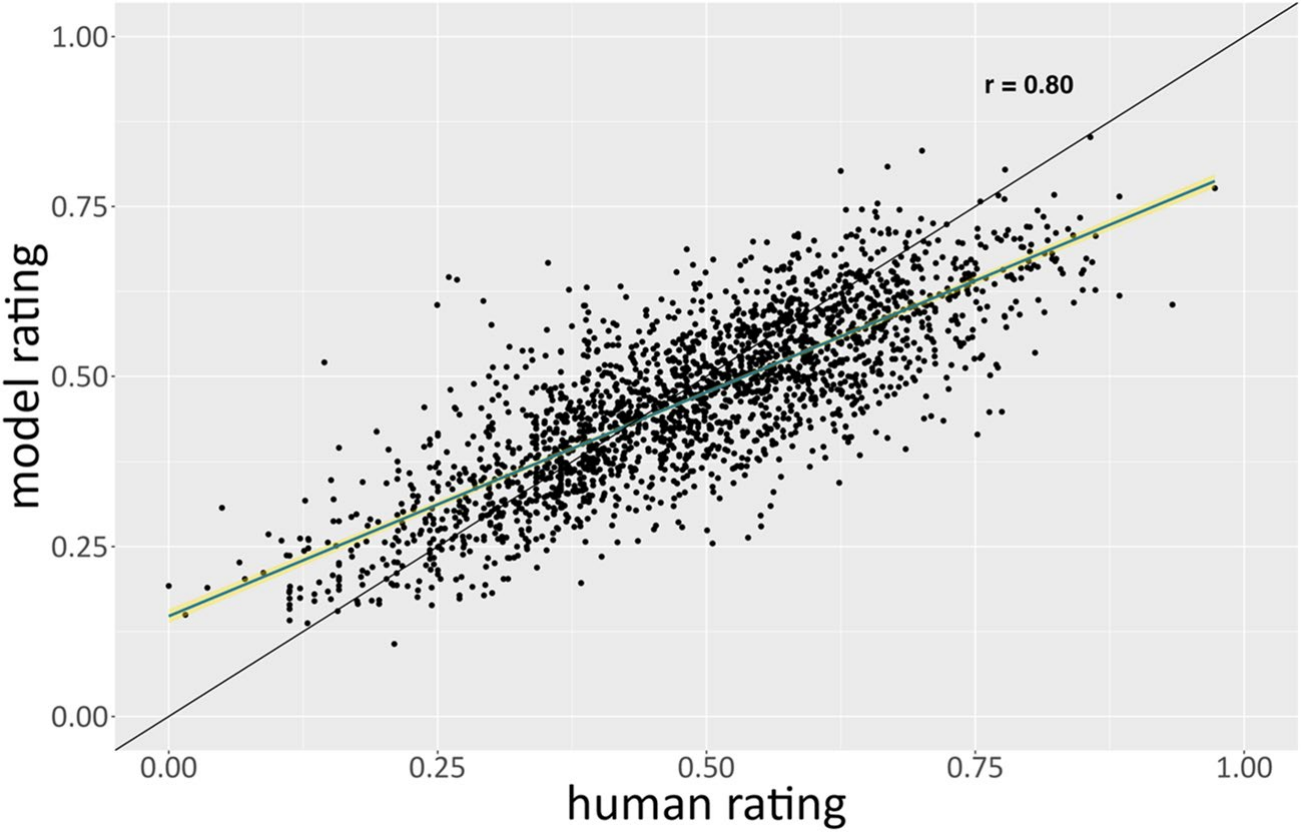
Test set: Predictive accuracy by rating magnitude. Each dot corresponds to a sketch from the test subset of the primary dataset. The y-axis shows the value predicted by AuDrA whereas the x-axis shows the creativity value provided by human raters. The black diagonal line reflects ideal performance (*r* = 1). The blue line reflects the line of best fit with 95%-confidence intervals in yellow

As with the validation set, we again qualitatively examined how AuDrA performed as a function of the drawings’ human-judged creativity. We found that AuDrA exhibited the same tendencies observed on the validation set. Generally, AuDrA was highly accurate but tended to underestimate the human evaluation of products that were most creative and overestimate the human evaluation of products that were deemed least creative (Fig. 5). Again, conditional rating reliability for the most and least creative products was lower than that of moderately creative products (Appendix Fig. 9), which may help account for poorer performance at the extremes in isolation or in conjunction with low item density in the training set (Appendix Fig. 10).

### Rater generalization analyses

In previous analyses, the validation and test subsets consisted of drawings AuDrA was never trained on but that were rated by the same pool of raters as the training set. We found that AuDrA can generalize effectively to new drawings, but we cannot generalize these findings to new raters it was not expressly trained with. The two rater generalization datasets (‘one’ and ‘two’) resulted from separate experiments, each of which consisted of over 600 drawings (and corresponding ratings) that were entirely independent from the training set. In this way, each of these datasets provides the opportunity to examine the extent to which AuDrA generalizes to both new drawings *and* new raters, which is important for the practical utility of this model in research applications.

**Fig. 6:**
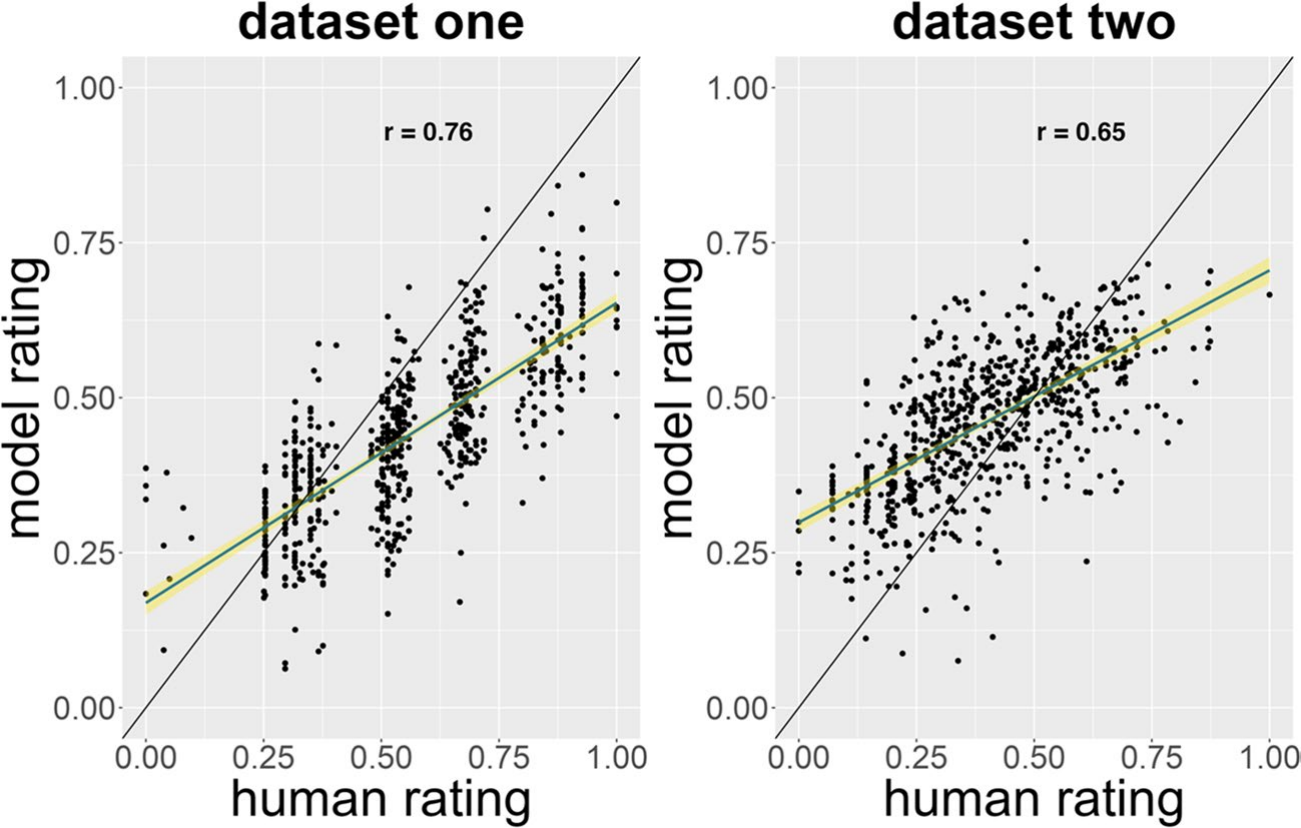
Rater generalization datasets: Predictive accuracy by rating magnitude. The datasets consisted of untrained sketches (i.e., unlike those in the validation and test sets), and were each rated by a pool of raters distinct from the primary dataset. In each panel, a dot corresponds to a specific sketch from its respective dataset. The *y*-axis shows the AuDrA-predicted creativity rating whereas the *x*-axis shows the rating provided by human raters. The black diagonal line reflects perfect performance (*r* = 1). The blue line shows the line of best fit and the yellow reflects 95% confidence intervals. Note that, due to the small number of raters (*n* = 3) and 1–5 response range, the data appear nearly ordinal in dataset one

Despite the fact that each of the rater generalization datasets was rather small and rated by a smaller set of raters (e.g., *n* = 3) relative to the primary dataset – a configuration that resembles typical experiments in creativity research in terms of number of raters and productions to be rated – AuDrA provided predictions that were highly aligned with human judgments of creativity. On both rater generalization datasets one (MSE = .034; *r*_*AuDrA*_ = .76; 95% CI [.73, .79]) and two (MSE = .022; *r*_*AuDrA*_ = .65; 95% CI [.61, .69]), AuDrA correlated strongly with human creativity judgments (Fig. 6). Importantly, AuDrA’s correlation with human ratings for each was well in excess of the correlations between elaboration and human ratings in both rater generalization datasets one (*r*_*Ink*_ = .55; 95% CI [.5, .6]) and dataset two (*r*_*Ink*_ = .33; 95% CI [.26, .39]). In terms of the distribution of prediction errors AuDrA made on these generalization datasets, AuDrA displayed a pattern comparable to that of the validation and test subsets. That is, although generally accurate, AuDrA tended to overestimate the least creative products and underestimate the most creative products – likely attributable in part to lower conditional reliability for ratings at the low and high end of the spectrum (Appendix Fig. 9) and few training examples at those extremes of the spectrum (Appendix Fig. 10).

### Task and rater (far) generalization analyses

**Fig. 7:**
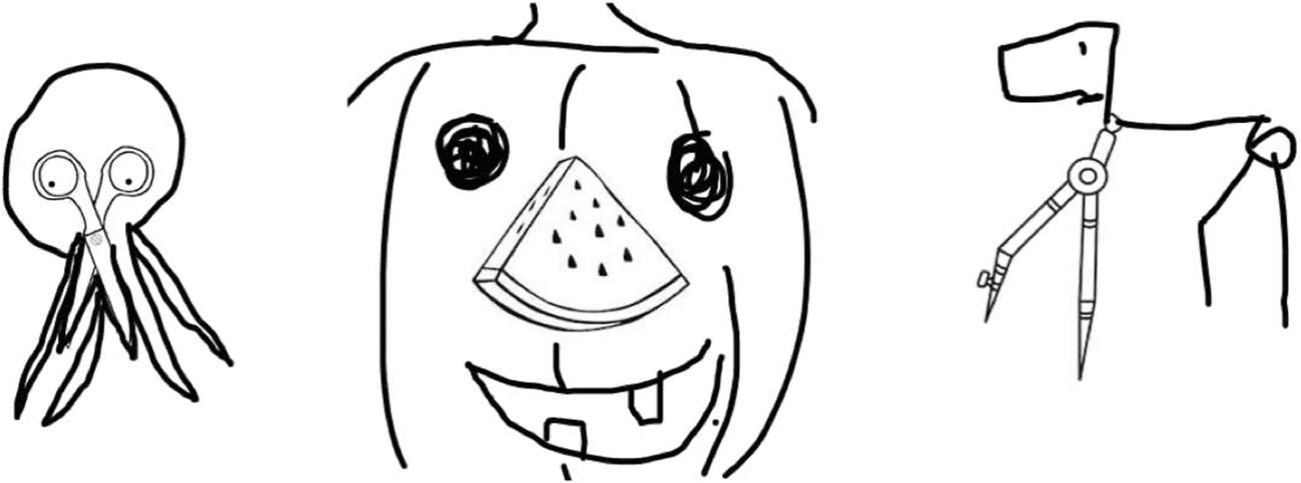
Examples responses from the MTCI object transformation task. From left to right; ‘scissors’, ‘watermelon’, and ‘compass’ items

Thus far, AuDrA has demonstrated the capacity to transfer to (1) new drawings it was never trained on and (2) new drawings it was never trained on that were rated by unique sets of raters it was never trained on. However, all drawings AuDrA has been tested on hitherto have been based on the “abstract” starting image task of Barbot’s (2018) structured drawing paradigm (MTCI “Incomplete shapes” task). The “concrete” version of Barbot’s drawing paradigm (MTCI “object transformation” task) involves providing participants with contour representations of real-world objects as the starting image for their drawings, instead of abstract starting images. Here, we use drawings from the concrete version of Barbot’s (2018) structured drawing task as a far generalization test of AuDrA’s performance. That is, in addition to testing AuDrA on drawings it was never trained on, rated by raters the model was not trained on, these drawings also pertained to a substantially different task than AuDrA was trained on (Fig. 7). Although the expectation was that AuDrA’s accuracy would be substantially decreased relative to the abstract starting image task it was trained on, this test served the important goal of gauging the boundaries of AuDrA’s predictive capabilities.

**Fig. 8:**
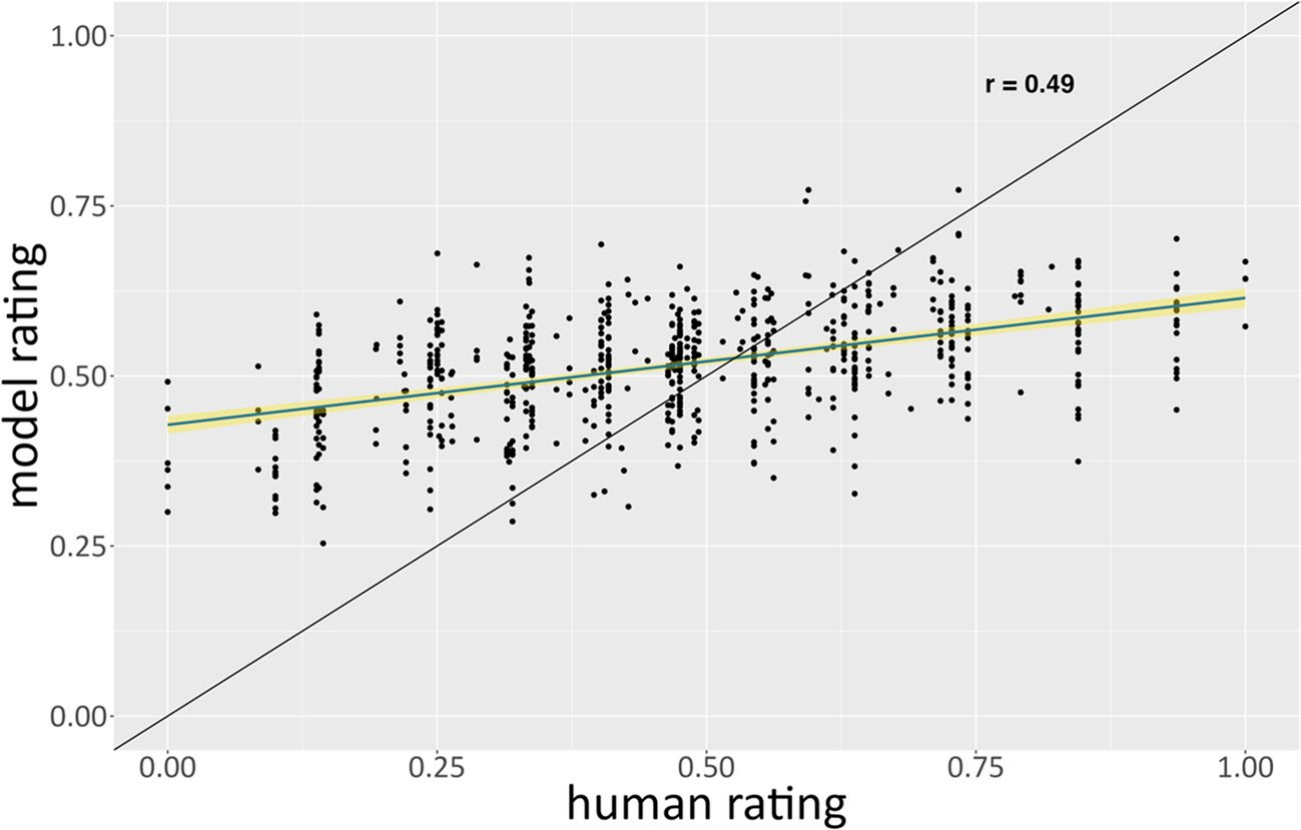
Far generalization set: Predictive accuracy by rating magnitude. The far generalization set contained drawings from distinct drawers, rated by distinct raters, and that came from a different task, relative to the training set. Each dot corresponds to a particular sketch. The *y*-axis shows the AuDrA-predicted creativity rating while the *x*-axis shows the rating provided by human raters. The black diagonal line reflects perfect performance (*r* = 1). The blue line shows the line of best fit and the yellow reflects 95% confidence intervals around that curve. Note that, due to the small number of raters (*n* = 3) and 1*-*5 response range, the data appear nearly ordinal

As expected, we found that AuDrA’s correlation with human ratings (*r*_*AuDrA*_ = .49; 95% CI [.43, .54]; Fig. 8) was considerably lower than the correlations observed for the abstract drawing datasets shown above. However, despite the degraded performance, AuDrA achieved reasonably good fit to human ratings (MSE = .037) and numerically outperformed the correlation between elaboration and human ratings (*r*_*Ink*_ = .40; 95% CI [.34, .46]) – albeit by a much smaller magnitude than those observed for the abstract starting image task it was trained on. This highlights the importance of using AuDrA on tasks it was explicitly trained on when predictive accuracy is the goal, but also the promise of AuDrA to model creativity ratings on other graphic tasks.

On this “concrete” object-based task, AuDrA also showed the same pattern of prediction error seen on the abstract “incomplete shapes” task – the magnitude of which may, again, be attributable to low conditional reliability of ratings at the high and low end of the spectrum (Appendix Fig. 9) or little training supervision on items at the extremes of the rating scale (Appendix Fig. 10). However, in contrast to AuDrA’s previously shown pattern of larger errors on the most creative products (relative to the least creative products), AuDrA showed larger errors on the *least* creative products on the object task. It is important to highlight that neural network behavior is a direct product of the training data it receives. Accordingly, abstract starting images that were turned into recognizable and/or closed figures may have been associated with higher creativity ratings during the learning process. If so, the object starting images might push AuDrA toward higher creativity ratings, even in cases where additional embellishment beyond the starting image is minimal.

## Discussion

Creativity research has benefited from the development of computational scoring methods, overcoming the burden of human rating, and offering increased standardization to the scoring process. Yet such developments have been restricted to verbal creativity tasks, with limited tools available for visual, or graphic, creativity tasks. In this work, we trained a deep convolutional neural network (ResNet) to automatically predict the creative quality of sketches on a psychometric test of visual creativity. We found that the model successfully learned to predict the creativity scores of human drawings it was not trained on with remarkably high accuracy. Table 1 summarizes properties of, and results for, each dataset. The model showed evidence of learning visual features of drawings, beyond simply predicting their level of elaboration (i.e., ink on the page). Critically, AuDrA generalized to predict the creativity of sketches from entirely independent datasets of human ratings that were not used in training the model. As expected, AuDrA showed poorer performance on a drawing *task* it was not trained on, pointing to a boundary condition of its predictive power. The present work represents the first extensively trained and vetted demonstration that machine learning methods can be reliably used to predict human creativity on drawing tasks, routinely used in creativity research. We provide open access to the AuDrA model with the goal of accelerating the pace of discovery in creativity research.

**Table 1:** Dataset characteristics and AuDrA results

Dataset	New task?	New drawing?	New raters?	Drawing	Raters	r	r_ink	ICC
Primary: validation	✗	✓	✗	1104	50	.81 [.79, .83]	.53 [.49, .57]	.9 [.89, .91]
Primary: test	✗	✓	✗	2216	50	.80 [.79, .82]	.41 [.37, .44]	.9 [.89, .9]
Rater generalization 1	✗	✓	✓	670	3	.76 [.73, .79]	.55 [.5, .6]	.73 [.69, .76]
Rater generalization 2	✗	✓	✓	722	6	.65 [.61, .69]	.33 [.26, .39]	.9 [.89, .91]
Rater + task generalization	✓	✓	✓	679	3	.49 [.43, .54]	.40 [.34, .46]	.63 [.58, .68]

*Note*: The New task/drawings/raters columns denote similarities and differences relative to the dataset AuDrA was trained on. The Drawings and Raters columns refer to the number of unique drawings and raters in each dataset, respectively. r reflects the Pearson correlation between AuDrA’s predictions and composite human ratings along with 95% confidence interval (in brackets). r_ink corresponds to the Pearson correlation between the elaboration metric and human ratings, with 95% confidence interval in brackets. Intraclass correlation coefficients (ICC) with 95% confidence interval (in brackets) were computed using a two-way random effects model for average consistency (ICC[C,k]) via the ‘irrNA’ package in R (Brueckl & Heuer, 2022). This variant of ICC is equivalent to Cronbach’s alpha

We obtained a large batch of drawings (*n* = ~ 11,000) and corresponding creativity ratings from human raters (*n* = 50 raters) to train and test the drawing prediction model. A computational experiment showed that the best performing model required 136 training epochs to learn to predict human creativity ratings, hitting a validation set model-human correlation of *r* = .81 – substantially exceeding the extent to which “ink on the page” (i.e., drawing elaboration) predicted creativity. Importantly, when tested on the larger held-out test set, the final model matched human ratings equally as well (*r* = .80), again surpassing ink on the page. Put another way, approximately 65% of the variance in human ratings could be explained by the model-predicted scores on the test set. Also of note is that AuDrA’s strong prediction of visual creativity was at the level of individual sketches, not at the aggregated level of participants (averaged across multiple drawings) which can cover up insensitivities to response-level variation. The strong prediction of creativity ratings, beyond the baseline of their elaborative quality (i.e., ink), suggests that AuDrA developed a sophisticated “understanding” of the features of drawings that humans find creative, and that it was not simply learning a “more is better” heuristic in predicting human creativity.

For reference, we compare the magnitude of AuDrA’s correlation to research on verbal creativity using semantic distance. In a recent study on creative writing, for example, Johnson et al. (2022) applied the BERT neural network model to automatically score creativity on short stories, finding that the best prediction of human ratings (across multiple tasks and samples) yielded a value of *r* = .76 (i.e., correlation between human ratings and a single short story) – comparable to the prediction magnitude found in one of the *rater* *generalization* samples in the present work (e.g., *r* = .76, dataset one). Notably, creative writing is actually an exception in the automated assessment literature: other studies on verbal creativity (e.g., divergent thinking tasks such as the Alternate Use Task, AUT) tend to show lower correlations at the response level (i.e., correlations between model scores and human ratings for single AUT responses; *r* ~ .3; Beaty & Johnson, 2021), with correlations only increasing once aggregated to the person level (i.e., across multiple responses; but see Yu et al., 2023 for improved performance at the response level).

AuDrA’s pattern of high correlations with human ratings across datasets and raters suggests that bottom-up variables explain much of the variance in humans’ perceptions of creativity (Lindell & Mueller, 2011). This follows from the nature of what CNNs are able to represent. CNNs only have the capacity to learn perceivable visual features – be they simple or complex – that are predictive of some outcome variable. However, they are not well equipped to represent latent relational/conceptual content that may elevate human evaluations of a given drawing (e.g., ‘this is a drawing of a couple holding hands in the rain’). The inability to acquire non-visuoperceptual knowledge may explain why AuDrA tended to underestimate the creativity of items that humans found most creative, particularly given the simplistic medium and constraints imposed by the starting images.

There may also be more general factors that contributed to AuDrA’s lower accuracy at the extreme ends of the creativity scale. CNNs are limited in their ability to learn visual features that predict some outcome when few examples of that outcome (i.e., rating magnitude) are present in the training dataset or when the outcome variable is noisy or unstable. Given the data occupied a relatively normal distribution (Appendix Fig. 10), the model received very little supervision at the high and low ends of the scale. Moreover, the conditional reliability at the ends of the scale was lower (Appendix Fig. 9), which added noise to the model’s supervision. It should be noted that conditional reliability at the scale’s extremes was much higher in the primary dataset relative to the smaller generalization sets. Thus, an important goal for future work is to enhance model performance at the extremes. In terms of minimizing noise in the training signal, future work should employ rater pools that are comparable in size, or larger, than the primary dataset in the present work. With respect to low data density at the tails of the rating distribution, future work may use compensatory methods to increase data density in the tails. One machine learning approach to address such imbalances is to use selective data sampling, such that the distribution of the outcome variable (in this case, creativity rating) in the training dataset is close to uniform. This can be done by randomly sampling only a subset of the high probability items each epoch and/or by duplicating low probability items for each training epoch (Van Hulse et al., 2007), and can be performed in conjunction with other data augmentation (e.g., vertically flipping the image). Future work should explore the potential for such techniques to improve AuDrA’s overall predictive accuracy.

A key difference between the present study and prior work on automated scoring of verbal creativity – beyond differences in response modality (visual vs. verbal) – is the nature of the model training involved. Because semantic distance methods rely on pre-trained or “out of the box” word embedding models that were not trained specifically to predict human creativity ratings, they represent “unsupervised learning” approaches (Zhou, 2021). The present work, in contrast, provided a CNN with accuracy feedback on its predictions (via the human-provided ratings); this feedback was used to update the weights of the model to optimize predictive accuracy for this specific drawing task and set of drawing prompts. Because human-provided ratings were used to update the model, the current work represents a “supervised learning” approach (for further discussion of supervised and unsupervised methods in creativity assessment see Buczak et al., 2023). Future studies on verbal creativity assessment may similarly benefit from supervised “fine-tuning” of large language models (e.g., BERT, GPT3) to maximize their prediction of individual verbal responses, which may boost the signal of creativity prediction at the response level (*r* ~ = .8; see Organisciak et al., 2023).

Another potential direction for future research is to combine verbal and visual information in the prediction of human creativity. Indeed, drawing tasks, such as the drawing task used in the present study, typically ask participants to provide a label or description for their drawing. Labels provide additional context for the human rating process, and they are aligned with the goal of drawing tasks in creativity assessment for expressing an original idea – not simply showing technical drawing proficiency (which is confounded with artistic expertise; Barbot, 2018). On the other hand, a strength of drawing tasks is that they have the potential to be less biased against participants with less language proficiency/vocabulary knowledge, potentially capturing a “purer” form of creativity that relies less on language and is less associated with education. Although the drawing labels were included with the drawings in the present study for the purposes of creativity rating – to clarify the drawings, which were often abstract and difficult to discern for human raters – we did not incorporate the labels when training the image-based machine learning model. One reason for not including the labels (in addition to CNNs being tailored to intake images, not text) was that the drawing datasets came from multiple countries, requiring a translation of the labels to English for the purpose of creativity scoring by our 50 English-speaking raters. Nevertheless, in our view, it is all the more remarkable that AuDrA could predict human ratings with only the visual information, whereas human raters were given both visual and verbal/conceptual information. Future studies might add verbal label knowledge (e.g., via transformer neural network embeddings; Vaswani et al., 2017) to aid creativity prediction of drawings, while considering and adjusting for the potential linguistic biases related to SES, education, and other experience that such labels may introduce.

Despite AuDrA’s impressive performance in predicting human ratings, we identified a limit of its predictive power. Specifically, AuDrA showed a substantial performance decline on drawing tasks that it was not trained on, i.e., the “concrete” object drawings task. This performance decrement was to be expected, and it is consistent with prior machine learning research showing limited far transfer to untrained tasks (e.g., von Rueden et al., 2023). Notably, however, AuDrA’s prediction of human ratings was still nominally higher than ink on the page, suggesting that it was able to detect some visual features predictive of ratings in the concrete object drawings, yielding a correlation in the range of what a human rater would obtain with a group of human raters. Future research could attempt to expand the range of drawing tasks predicted by AuDrA by specifically retraining the model on additional tasks and ratings. AuDrA is openly available for such purposes, and we hope it will serve as a benchmark for future work to build upon.

We see many possible applications of AuDrA for creativity researchers. For example, the model could be used as a form of dynamic feedback, generating real-time creativity predictions as people iteratively produce new sketches. Real-time drawing feedback could accelerate the learning process for interventions aimed at improving creative thinking. In addition, AuDrA could be applied at longer timescales in the context of longitudinal studies, to assess developmental trajectories of visual creativity and to track the efficacy of creativity interventions. And there is still much to learn about what AuDrA itself is learning about human creativity. While the present work makes abundantly clear that it does not simply boil down to a ‘more’ rule, understanding the exact nature of what’s creative to humans through the learned model’s lens is a vital avenue for future work. Aesthetics research could further explore the visual features of the input images that are predictive of human ratings and those predicted by the model (e.g., curvature, symmetry). Complementary computational approaches to visualizing feature representations (e.g., Olah et al., 2017) may be used to peer into the figurative ‘black box’ and shed light on features, textures, and objects the trained model (and consequently humans) find creative.

As one last direction for future work, we note the breakneck speed at which machine learning advances are made. In the current work, we drew on the CNN framework as the computer vision backbone of AuDrA. While we selected ResNet to serve that role in the present work, there exists a constellation of other CNNs, with different design principles, that may be explored and compared to our approach. Additionally – and in contrast to the CNN framework used here – the transformer framework (Dosovitskiy et al., 2021; Vaswani et al., 2017), which incorporates sophisticated attention mechanisms that allow the model to learn contextually sensitive relations between parts of the input (i.e., text or images) provides another promising avenue for further performance gains.

## Figures and Tables

**Fig. 9:**
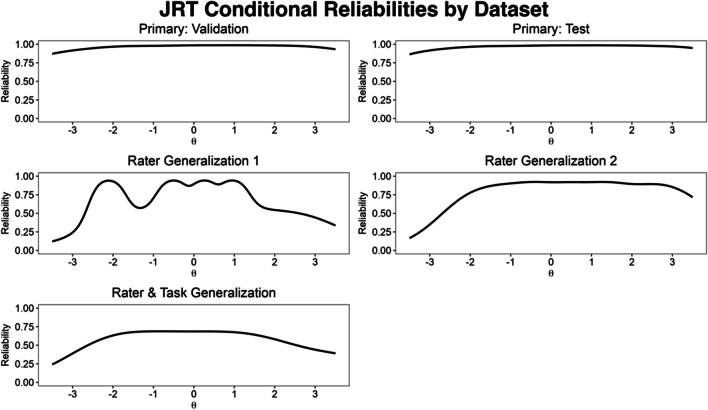
Conditional reliabilities across degrees of creativity (i.e., JRT theta) for each of the datasets used to test AuDrA. Lower theta values correspond to lower creativity, and vice versa

**Fig. 10:**
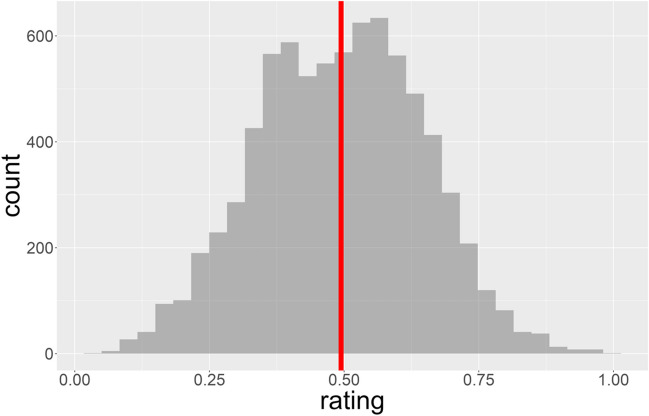
Histogram showing the counts of items in each of 30 rating bins. The red line represents the mean rating (.492); the median item’s rating was roughly equivalent (.497). The rating values correspond to JRT theta values normalized to the 0-1 scale AuDrA was trained on

## Appendix 1 Hyperparameter search: Top five settings

The hyperparameter search yielded several settings that performed roughly the same, as measured by mean squared error at the conclusion of the 150-epoch training period. These top settings varied with respect to model depth, ImageNet pre-training, and learning rate magnitude. Though regularities are hard to discern, it is notable that all but one of the top five settings used a batch size of 16 images.

**Table 2:** The top five hyperparameter search fits to the validation dataset. MSE refers to mean squared error. The table is ordered by MSE, from smallest (best performance) to largest. The top row contains the model settings used for the test and generalization datasets in the manuscript

MSE	Model Depth	Pre-trained?	Learning Rate	Batch Size
.002305	resnet18	True	.000347	16
.002697	resnet18	False	.000027	32
.003048	resnet34	True	.002809	16
.003100	resnet34	True	.000020	16
.003974	resnet18	False	.001441	16

## Appendix 2 Judge response theory conditional rating reliabilities by dataset

The conditional reliabilities show, in general, that items towards the extremes of creativity (i.e., least or most creative) have lower reliabilities than their moderate creativity counterparts. This may be due in part to the relatively low number of items at those extremes. However, the large number of raters used for the primary dataset (50 raters) appears to attenuate the sharp drop off in reliability observed in the other datasets, which employed either three or six raters. Reliability for products deemed least creative is generally lower than products deemed most creative by raters.

## Appendix 3 Distribution of ratings in the training dataset

The ratings the model was trained on assumed a roughly Gaussian normal distribution, with most of the items receiving middling ratings (i.e., near the center of the rating scale) and relatively few items receiving ratings in the tails. As such, AuDrA received few learning experiences at the tails of the distribution.
